# Preliminary Results of an Astri/UWM EGNSS Receiver Antenna Calibration Facility

**DOI:** 10.3390/s21144639

**Published:** 2021-07-06

**Authors:** Karol Dawidowicz, Jacek Rapiński, Michał Śmieja, Paweł Wielgosz, Dawid Kwaśniak, Wojciech Jarmołowski, Tomasz Grzegory, Dariusz Tomaszewski, Joanna Janicka, Paweł Gołaszewski, Bogdan Wolak, Radosław Baryła, Grzegorz Krzan, Katarzyna Stępniak, Grec Florin-Catalin, Karol Brzostowski

**Affiliations:** 1 Institute of Geodesy and Civil Engineering, Faculty of Geoengineering, University of Warmia and Mazury, Oczapowskiego 1, 10-719 Olsztyn, Poland; jacek.rapinski@uwm.edu.pl (J.R.); pawel.wielgosz@uwm.edu.pl (P.W.); dawidkwachu@wp.pl (D.K.); wojciech.jarmolowski@uwm.edu.pl (W.J.); dariusz.tomaszewski@uwm.edu.pl (D.T.); joanna.janicka@uwm.edu.pl (J.J.); pawel.golaszewski@uwm.edu.pl (P.G.); bogdan.wolak@uwm.edu.pl (B.W.); radoslaw.baryla@uwm.edu.pl (R.B.); grzegorz.krzan@uwm.edu.pl (G.K.); katarzyna.stepniak@uwm.edu.pl (K.S.); 2 Department of Mechatronics and Technical-Computer Education, Faculty of Technical Sciences, University of Warmia and Mazury, Oczapowskiego 11, 10-710 Olsztyn, Poland; smieja@uwm.edu.pl; 3 Astri Polska, Krakowska 110/114, 02-256 Warsaw, Poland; Tomasz.GRZEGORY@astripolska.pl (T.G.); Karol.BRZOSTOWSKI@astripolska.pl (K.B.); 4 Directorate of Navigation, European Space Technology and Research Centre, European Space Agency, Postbus 299, 2200 AG Noordwijk, The Netherlands; Florin-Catalin.Grec@esa.int

**Keywords:** GNSS, antenna calibration, PCC

## Abstract

In 2019, the University of Warmia and Mazury in Olsztyn, in cooperation with Astri Polska, started a European Space Agency (ESA) project. The purpose of the project is the development and implementation of a field calibration procedure for a multi-frequency and multi-system global navigation satellite system (GNSS). The methodology and algorithms proposed in the project are inspired by the “Hannover” concept of absolute field receiver antenna calibration; however, some innovations are introduced. In our approach, the antenna rotation point is close to the nominal mean phase center (MPC) of the antenna, although it does not coincide with it. Additionally, a National Marine Electronics Association local time zone (NMEA ZDA) message is used to synchronize the robot with the GNSS time. We also propose some modifications in robot arm movement scenarios. Our first test results demonstrate consistent performance for the calibration strategy and calibration procedure. For the global positioning system (GPS) L1 frequency, the calibration results show good agreement with the IGS-type mean values. For high satellite elevations (20°–90°), the differences do not exceed 1.5 mm. For low elevation angles (0°–20°), the consistency of the results is worse and the differences exceed a 3 mm level in some cases.

## 1. Introduction

**Figure 1:**
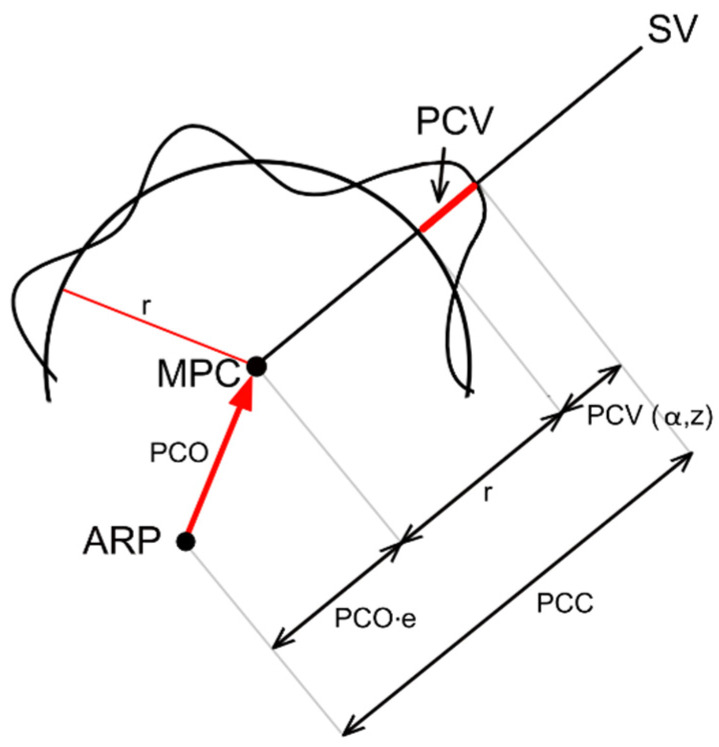
PCC as a function of PCO and PCV.

The phase centers of global navigation satellite system (GNSS) antennas (receivers and satellites) show biases that should be modeled for precise positioning applications [1,2,3]. The real antenna phase center is not fixed, but its location varies depending on the receiving signal’s direction and frequency. For modeling purposes, some auxiliary points, scalars, and vectors may be defined (Figure 1):

− The mean phase center (MPC) is defined as the point at which the signal phase has the smallest phase center variation (PCV);
− the antenna reference point (ARP) is defined by the International GNSS Service (IGS) as the intersection of the vertical symmetry axis of the antenna with its bottom plane;
− the antenna phase center offset (PCO) is a vector connecting ARP and MPC;
− r is the radius of a perfect phase sphere originating from MPC;
− PCV is a difference between the instant position of the antenna phase center and perfect phase sphere originating from MPC in the satellite vehicle (SV) position function. The combined phase center correction (PCC) is a function of PCO and PCV (1).

PCC(α,z) = r + PCO·*e* + PCV(α,z) (1)

In Equation (1), α and z are the horizontal and zenithal angles of the SV position in the antenna body frame and *e* is the line of sight unit vector.

The GNSS antenna PCV is still important issue in many various applications [4,5,6,7,8]. Accurate PCCs are a key element, especially in field of geodesy, where GNSS measurements are used to establish reference frames [9,10]. The results of many studies [1,11,12,13] prove that the omission of antenna calibration correction or use of incorrect correction significantly affects results. In [1,12], the authors demonstrated the differences in position results as caused by using mean calibrations instead of individual calibrations, where the differences can reach up to 1 cm in the up component and for the horizontal components are generally below 4 mm. In [13], the latest changes, improvements, and deficiencies of PCC models were presented. Appropriate PCCs are also important for various geophysical applications [14,15,16,17]. One such application is precise time and frequency transfer and intercontinental and continental links for different metrology sites [14,15]. Accurate antenna models also play an essential role in meteorology for the estimation of GNSS based troposphere zenith delays (ZTDs) [16,17].

So far, absolute GNSS antenna models have been created using robot field calibration for two frequencies, i.e., global positioning systems (GPSs) and Globalnaya Navigazionnaya Sputnikovaya Sistema (GLONASS) systems. Besides the GPS and GLONASS systems, two other systems are approaching full operational capability. The European Union (EU), with the help of the European Space Agency (ESA), has introduced the Galileo positioning system. At approximately the same time, China has developed the Beidou system (BDS). Additionally, existing satellite navigation systems have evolved into new modernized forms. Modernized GPS and GLONASS systems transmit new signals using new carrier frequencies. The modernized GNSS satellites will broadcast at least three civil signals in a range of frequency bands. The development of new satellite systems and the introduction of new carrier frequencies to existing ones results in the need to include them in antenna calibration procedures.

Recently, Geoscience Australia (GA) became one of the six GNSS antenna calibration facilities in the world and is the only one situated in the southern hemisphere [18]. The other five are the National Geodetic Survey (NGS) (Virginia, USA), University of Bonn (UB) (Bonn, Germany), SenB (Berlin, Germany), Geo++ (Hannover, Germany), and the Institute of Geodesy (IfE) (Hannover, Germany) facilities. The first results obtained by two new calibration facilities at Wuhan University [19] and Eidgenössische Technische Hochschule (ETH) Zürich [20,21] have recently been published.

## 2. Antenna Calibration Techniques

Methodologies for calibrating GNSS antennas have been developed since the 1980s. So far, different antenna calibration techniques have been established. Generally, they can be divided into absolute and relative or field and laboratory procedures [22]. Relative PCCs are created when a calibrated GNSS antenna phase center position is measured relative to a reference antenna (typically Dorne Margolin T, assuming that the reference antennas PCVs are zero). Absolute methods include scenarios where PCVs are determined independently of the reference antenna during calibration. A field procedure is based on real satellite signal tracking [23,24]. A laboratory procedure is carried out in an anechoic chamber and uses simulated GNSS signals [3].

Besides classification based on the calibration method, PCC models are categorized into individual and mean modes. An individual antenna PCC model is created when a specific antenna/radome is calibrated. In mean-type calibration, several antennas/radomes of the same type are calibrated and the final PCC model is created through the combinations of all results into single individual file.

The absolute field calibration procedure developed in Hannover by IfE and Geo++ [25] uses a robot that tilts and rotates the antenna. In this methodology, homogeneous coverage of observation is ensured, even for negative elevations, and real satellite signals are tracked during the calibration. The majority of errors and biases are mathematically canceled by using a short base (<10 m) during calibration and observing the creation of time differences of single difference (TDSD).

Nevertheless, GNSS antenna calibration is still a challenging issue. Depending on the method, the GNSS antenna PCCs may differ in the order of several millimeters [1,2,23,24]. In [23], the results of absolute calibration provided by three calibration centers (two in-field and one in an anechoic chamber) were compared (Geo++, NGS, and University of Bonn). The results proved that the differences in PCCs can reach up to 5 mm in the case of low elevation corrections (up to 20°). Consequently, the University of Warmia and Mazury in Olsztyn, in cooperation with Astri Polska, started a project aimed at the development and implementation of a receiver antenna field calibration facility for a multi-frequency and multi-system GNSS. The applied approach is based on the “Hannover” idea [24,25,26], where the PCO and PCV results of an absolute test antenna are determined by the analysis of the observable TDSD (one satellite only).

In comparison to the “Hannover” concept, some innovations are introduced with our approach. The first innovation is a different antenna rotation point definition. In the “Hannover” concept, during GNSS antenna calibration, the antenna is rotated around a fixed point, i.e., a nominal phase center, which is usually a GPS L1 frequency MPC. As MPCs are different for different antennas and frequencies, we decided to define our antenna rotation point as fixed for all calibrations, regardless of the antenna type or signal frequency. As a result, we have the same stable fixed rotation point in each calibration, but the antennas are not rotating around their nominal phase center. Appropriate corrections should be applied in order to achieve the effect of rotating around a nominal phase center. These corrections, which are the function of difference between the nominal phase center for a specific frequency and the rotation point position, as well as the SV position, are calculated during a post-processing stage.

**Figure 2:**
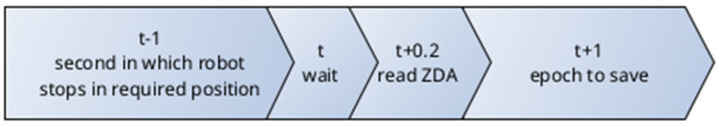
Robot vs. GNSS time synchronization.

One of the crucial issues in a PCV determination procedure is robot and GNSS time synchronization. The tilt and rotation angles must be recorded with a GNSS timestamp. To achieve this synchronization, a National Marine Electronics Association local time zone (NMEA ZDA) message containing a coordinated universal time (UTC) timestamp transmitted by one of the receivers was proposed. This message appears about 20 ms after a full GPS second. The robot’s controller, together with a PLC (programmable logic controller), captures this time and waits until the beginning of following second to ensure time synchronization (Figure 2). After this period, the timestamp and current antenna orientation are recorded for post-processing. The communication between the receiver, PLC, robot controller, and personal computer (PC), which is used as a data storage device, is performed with the aid of an ethernet transmission control protocol (TCP).

We also propose some modifications in robot arm movements scenarios. In the “Hannower” concept, the measuring program is satellite constellation-dependent and the average total number of different orientations in one calibration procedure is between 6000 and 8000 [27]. In our approach, we propose a regular robot arm movement scenario which is not dependent on any satellite constellation. Our main robot arm unit (RAU) movement scenario is based on a changing position for the zenith angle in the range from 0° to 60° in 5° steps and a changing position for the azimuth angle in the range from 0° to 350° in 10° steps. The use of these algorithms is very easy, and, on the other hand, they allow us to collect sufficient data for one calibration (in the case of a full satellite constellation) within about 1 h and 40 min. Our procedures have been extensively tested and this paper presents the initial results for an Astri/UWM calibration facility with the GPS L1 frequency.

## 3. GNSS Receiver Antenna Calibration Facility

A calibration system can be divided into two main blocks, namely, hardware and software. The main hardware components include an industrial robot, external oscillator, antennas and receivers, robot mounting set, and a personal computer with appropriate wiring. The software consists of the robot motion and control software, data collection software, and PCC modeling software.

**Figure 3:**
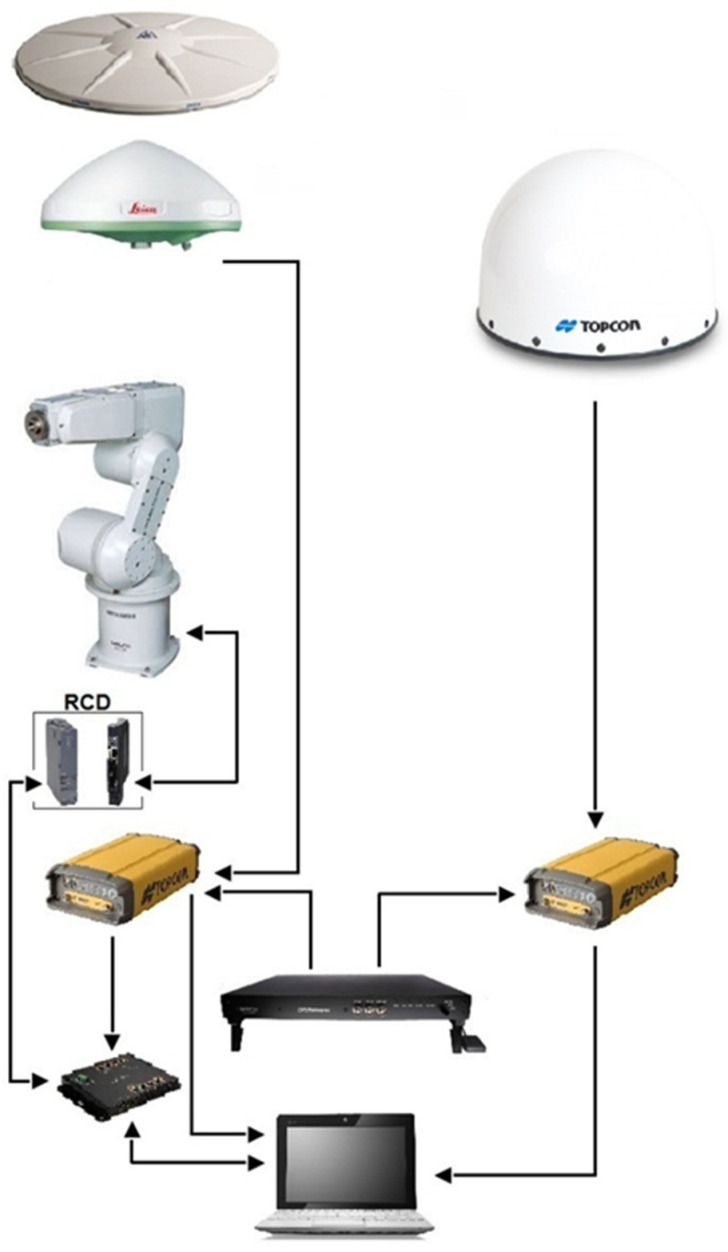
The hardware part of the GNSS antenna calibration system.

The system design of the proposed method for the absolute field GNSS antenna calibration is explained in Figure 3. On the short baseline (~5 m), the reference antenna and test antenna (mounted on the robot) are connected to the receivers and linked to the external frequency standard. Connecting to an external frequency standard is crucial for the reduction of individual receiver clock errors. A PC is used to acquire raw GNSS data and keep control of the robot (initialization and control robot positioner). A robot control device (RCD) is used to control the robot movements, where the two main parts are the PLC and control unit (CU).

The antenna that is to be calibrated is mounted on the robot arm and then tilted and rotated around a fixed point The optimal duration of a single robot arm movement appropriate to obtain two successive positions is 1–5 s. A full set of such movements will result in precise GNSS measurements covering the entire antenna hemisphere.

## 4. Algorithm and Strategies of Data Processing

Two main steps can be distinguished in PCC modeling. The first is the data preparation step, where data are corrected, i.e., other error and bias sources are reduced or eliminated. The second is the solution step, in which corrections are modeled. Several known factors and biases must be addressed to prepare raw carrier phase data for PCC estimation. Undifferenced phase observation of $\Phi_{f_{A}}^{j}$ [m] is used in this case for the frequency *f* from station A to satellite *j*.

(2) $$\Phi_{f_{A}}^{j}=\rho_{A}^{j}+c\left(\delta t_{A}-\delta t^{j}\right)+\lambda_{f}N_{f_{A}}+\left(\delta \varphi_{f_{A}}-\delta \varphi_{f}^{j}\right)+T_{f_{A}}^{j}-I_{f_{A}}^{j}+MP_{f_{A}}^{j}+PCV_{fA}+\varepsilon_{f_{A}}^{j}$$

The equation considers the geometric distance $\rho_{A}^{j}$, the speed of light *c*, the receiver and satellite clock errors $\delta t_{A}$ and $\delta t^{j}$, respectively, the tropospheric $T_{f_{A}}^{j}$ delay and ionospheric $I_{f_{A}}^{j}$ delay, the ambiguity term $N_{f_{A}}$, the hardware delays in the receiver ($\delta \varphi_{f_{A}}$) and satellite ($\delta \varphi_{f}^{j}$), the phase multipath term $MP_{f_{A}}^{j}$, the phase center variations $PCV_{fA}$, and measurement noise $\varepsilon_{f_{A}}^{j}$.

By forming single differences $SD_{A,B}^{j}\left(t_{i}\right)$ on a short baseline of approximate 5 m between stations A and B at epoch $t_{i}$, the effects introduced by the satellite and by distance-dependent factors are nearly eliminated.

(3) $$SD_{A,B}^{j}\left(t_{i}\right)=\Phi_{f_{A}}^{j}-\Phi_{f_{B}}^{j}$$

**Figure 4:**
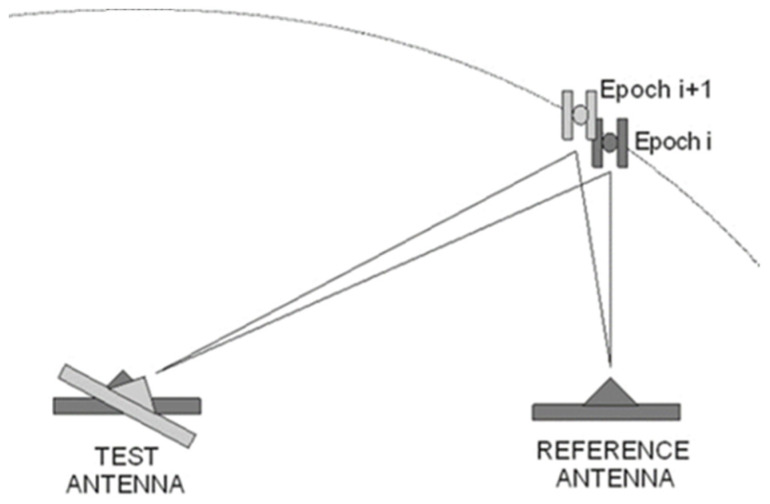
The idea of TDSD for GNSS receiver antenna calibration purposes.

One may apply time differences (Figure 4) to single differences from Equation (3).

(4) $$\Delta SD_{f_{A,B}}^{j}\left(t_{i}\right)=SD_{f_{A,B}}^{j}\left(t_{i+1}\right)-SD_{f_{A,B}}^{j}\left(t_{i}\right)$$

We can obtain access to the observation equation for any given frequency:

(5) $$\Delta SD=c\Delta \delta_{A,B}\left(t_{i},t_{i+1}\right)+\Delta PCV_{A}^{j}\left(t_{i},t_{i+1}\right)+\Delta MP_{A,B}^{j}\left(t_{i},t_{i+1}\right)+\Delta d_{A,B}^{j}\left(t_{i},t_{i+1}\right)+\;\Delta T_{A,B}^{j}\left(t_{i},t_{i+1}\right)-\Delta I_{A,B}^{j}\left(t_{i},t_{i+1}\right)+\Delta \rho_{A,B}^{j}\left(t_{i},t_{i+1}\right)+PWU_{A,B}^{j}\left(t_{i},t_{i+1}\right),$$

The equation includes the differential receiver clock error $c\Delta \delta_{A,B}\left(t_{i},t_{i+1}\right)$, topography of the PCV pattern $\Delta PCV_{A}^{j}\left(t_{i},t_{i+1}\right),$ and additional differential error terms. As the time offset between consecutive epochs is very short (within 5 s), and the station baseline is also very short (within 10 m), the terms $\Delta T_{A,\;B}^{j}\left(t_{i},t_{i+1}\right)$ and $\Delta I_{A,B}^{j}\left(t_{i},t_{i+1}\right)$ are neglected. The ambiguity term and the PCV are eliminated, as well as a multipath term at station B. The methodology also significantly reduces multipath effects at station A. As the PCVs of the calibrated antenna are determined by changing the orientation between subsequent epochs, the phase wind up correction $PWU_{A,B}^{j}\left(t_{i},t_{i+1}\right)\;$has to be introduced to Equation (5).

In the solution step, corrections are evaluated in two stages, namely, MPC modeling followed by PCVs modeling. The input data are TDSDs after error and bias cleaning. The MPC is determined using the assumption that $\sum^{\;}{\left(PCV\right)}^{2}=min.\;$As a result, ΔN, ΔE, and ΔU of MPC will be obtained. In the next step, the input data are PCV residuals obtained after MPC determination. PCVs are modeled using spherical harmonics. At least three independent solutions are provided, and then PCVs in an elevation-only function are compared and finally the most similar solutions are selected to calculate the final mean solution. The final step is achieved by converting the achieved results to the ANTEX format.

In standard analysis, PCVs are parametrized with spherical harmonic expansion (SHE) using an expansion of degree of n = 8 and an order m = 5. Without the loss of generality and the consistent comparison of different PCVs, their values must be transformed into one common PCO and then re-centered at the zenith to zero.

## 5. Data Collection

**Figure 5:**
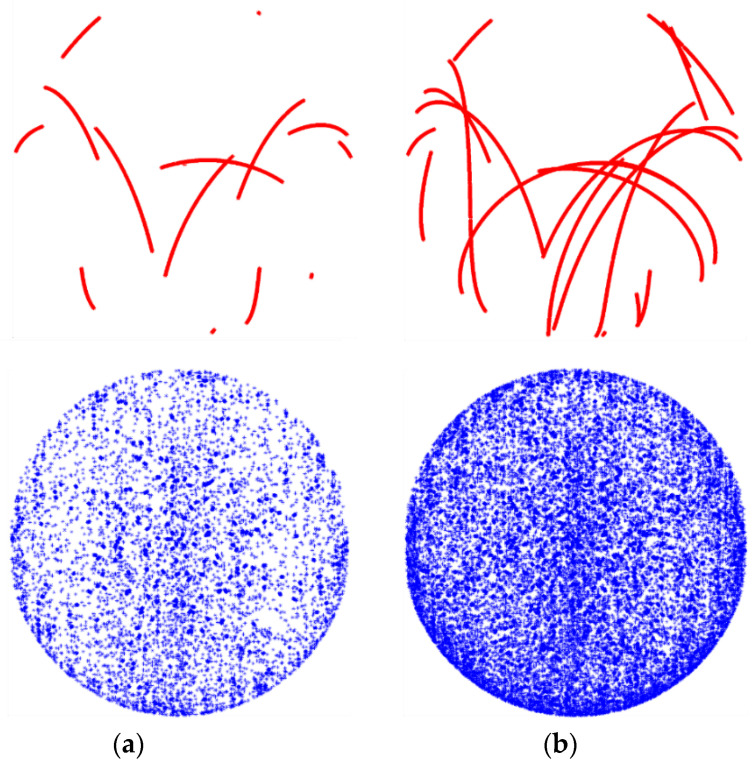
Observations coverage on test antenna hemisphere in case of static antenna (red) and robot rotated and tilted one (blue): (**a**) after 1 h 40 min (1 calibration); (**b**) after 5 h (3 calibrations).

To obtain a precise and reliable PCC model, it is critical to distribute the observations over the entire antenna’s hemisphere in a dense and uniform manner. Some additional observations are also recommended for negative elevation angles. Meeting these requirements would not be possible without a high-precision robot that can flexibly rotate and tilt the antenna during the calibration process. In the Astri/UWM calibration procedure, it takes less than 2 h to collect the required number of observations, and, in consequence, to derive a PCC model from one calibration session. To verify the obtained results and determine the model’s internal accuracy, it is recommended to carry out at least three calibration sessions, which altogether takes about 5 h (Figure 5).

Figure 5 presents coverage of observations after 1 h and 40 min of observation (1 calibration) and after 5 h of observations (3 calibrations). The red dots indicate observations in a static mode (without antenna tilting and rotating). It is thereby clear that even long static measurements cannot provide appropriate coverage of the observations. Only the use of the robot together with the proper rotation scenario ensures sufficient number of observations for calibration purposes (blue dots in Figure 5).

**Figure 6:**
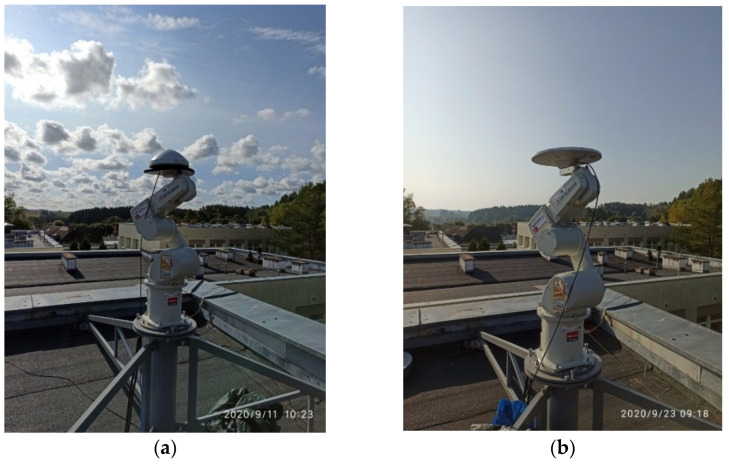
The antennas during calibration: (**a**) LEIAR10/NONE; (**b**) TRM115000.00/NONE.

In this paper, the results of the initial calibration experiments carried out to verify the applied algorithms and the implemented calibration procedure are presented. Two models of GNSS antennas were chosen to validate the obtained PCCs models, namely, LEIAR10/NONE and TRM115000.00/NONE (Figure 6). Several institutes have previously calibrated these antennas and the results have been published in the igs05.atx, igs08.atx and igs14.atx files of the IGS (https://ftp.epncb.oma.be/ftp/station/general/igs14.atx; access date: 5 July 2021).

Two experiments were conducted using the two aforementioned antenna types in order to test the repeatability of calibration results and determine the external accuracy of the generated models in comparison to the results available in the igs14.atx file. Both antennas were calibrated in three sessions using 1-s measurement intervals. The data collection for each experiment was completed after reaching full observational coverage for the test antenna in three calibration sessions (about five hours). Detailed information concerning the calibration measurements can be found in Table 1.

As seen in Table 1, during both calibrations, we used the same type of receiver (Topcon Net-G3A) on both stations (reference robot), as recommended by the currently operating calibration facilities. Moreover, we used the same types and lengths of the wires for connecting receivers with the external clock and antennas to minimize the hardware delay effect.

## 6. PCO Accuracy Validation

The accuracy and stability of the obtained models can be validated by checking the reproducibility of the obtained results and comparison to existing models (igs14.atx). The results obtained for PCOs from the three calibrations, presented in Table 2, show that there were some slight deviations between different session results, which is reflected in standard deviations of 0.19 mm, 0.25 mm, and 0.56 mm in the northern, eastern, and vertical components, respectively, for the LEIAR10/NONE antenna. For the TRM115000.00/NONE antenna, the standard deviations reached 0.26 mm, 0.22 mm, and 0.52 mm in the northern, eastern, and vertical components, respectively. Generally, as shown in Table 2, both tested geodetic antennas (TRM115000.00/NONE and LEIAR10/NONE) show good result reproduction. The standard deviations for the northern and eastern components were smaller than 0.3 mm. For the vertical component, we achieved standard deviations that were slightly larger, reaching almost 0.6 mm.

In comparison to the igs14.atx values (Table 2), it can be seen that differences do not exceed 0.6 mm, 0.5 mm, and 2.0 mm in the northern, eastern, and vertical components, respectively, for the LEIAR10/NONE antenna. In the case of the TRM115000.00/NONE antenna, the differences do not exceed 0.4 mm, 0.9 mm, and 1.7 mm in the northern, eastern, and vertical components, respectively. Such differences are acceptable and often occur when individual and mean-type calibration results are compared [28,29].

## 7. Elevation-Only PCC Validation

PCCs are a function of the PCO and PCVs and can be expressed as elevation-only dependent or elevation- and azimuth-dependent functions (full PCCs). The first solution (elevation-only model) is usually used in so called “commercial software”, where the highest accuracy and precision of results is not required, and also in kinematic applications, where the horizontal orientation of a rover antenna cannot typically be measured.

**Figure 7:**
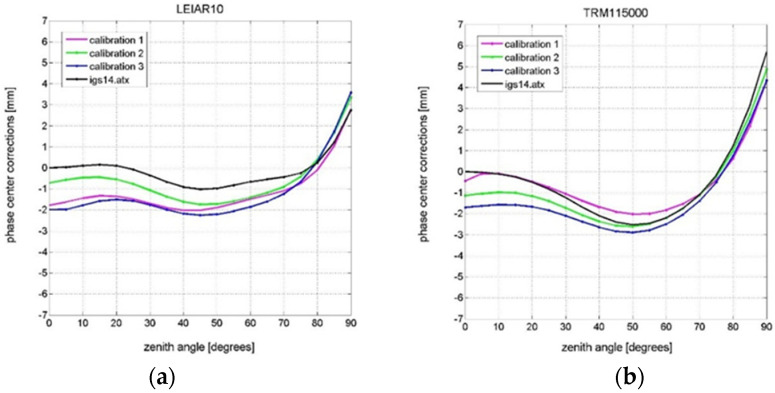
Zenith-dependent PCVs of the GPS L1 frequency: (**a**) LEIAR10/NONE antenna; (**b**) TRM115000.00/NONE antenna.

In this section, we analyze elevation-only dependent PCCs models obtained during Astri/UWM calibrations. At the first stage, both compared sets of results have to be shifted to PCV(α,0) = 0 (zenith PCV = 0) in case this was not done during the calibration process. This shift can be done by adding constant value δ=−PCV(α,0) to all derived PCVs. After adding δ, the PCO vertical component should also be corrected. The corrected PCO vertical component can be described in the form of $PCOup^{corr}=PCOup-\delta .$ In the final step, we can compare two models by reducing the PCCs of the first one (compared) to the PCO of the second one (reference) using a general formula [30]:

(6) $$PCC\left(\alpha ,z\right)=s^{T}PCO_{r}+\left(PCV_{c}\left(\alpha ,z\right)+s^{T}\left(PCO_{c}-PCO_{r}\right)\right)$$

where PCC denotes the compared PCC after reduction to reference PCO, PCO_r_ is the PCO of the reference model, PCV_c_ denotes the PCV of the compared model and PCO_c_ is the PCO of compared model. The resulting reference and compared PCVs can be analyzed by forming difference patterns (dPCCs). Figure 7 demonstrates the consistency between the calibrated results and their discrepancies with IGS-type (mean) values for both tested antennas.

It can be concluded from the analysis of the internal accuracy of the achieved results (PCCs model repeatability) that we have obtained consistent results. The differences do not exceed 1.5 mm in the whole estimated zenith angle range (from 0° to 90°) for both tested antennas. When we compare the Astri/UWM results with the reference model (igs14.atx), the differences increase slightly (e.g., calibration 3 for TRM115000.00/NONE antenna), exceeding 1 mm for the zenith angle regions.

**Figure 8:**
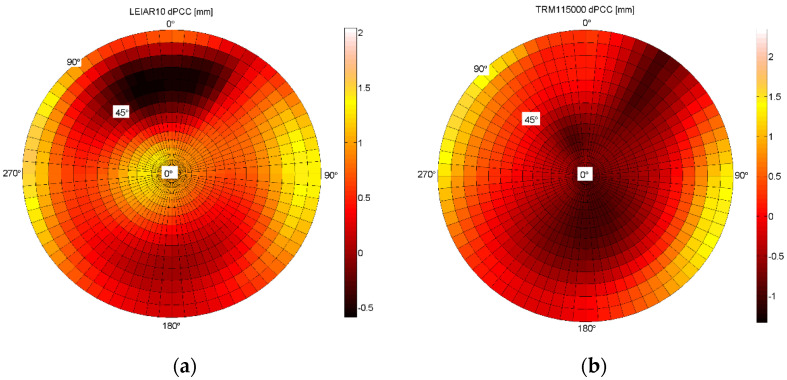
Differences between two independent calibrations of full PCCs for the GPS L1 frequency: (**a**) LEIAR10/NONE (left) antenna; (**b**) TRM115000.00/NONE (right) antenna.

The most significant differences occur at low zenith angles (from 0° to 30°), which is mainly caused by the differences in PCO vertical components. Some increases in differences can be also observed for very high zenith angles (from 80° to 90°), which can also be observed for full PCC comparison (see Figure 7 and Figure 8). In this case, the reason is higher noise in observations, which is common for high zenith angle signals [31].

## 8. Full PCCs Validation

The final step of Astri/UWM result assessment is the consideration of the full PCC model and accuracy validation. Validation can be performed as previously described by checking the reproducibility of the obtained results, as well as by comparison to existing models (e.g., igs14.atx). Figure 8 presents the differences between two independent calibrations carried out at an Astri/UWM calibration facility. For the GPS L1 frequency, correction comparison was performed using the aforementioned approach proposed by Schön and Kersten [30].

From the analysis of the internal accuracy of Astri/UWM results for the full PCCs model, it can be confirmed that we have obtained consistent results. The differences do not exceed 2 mm over the whole antenna hemisphere, and this is valid for LEIAR10/NONE, as well as for TRM115000.00/NONE antenna.

As for the elevation-only models, the most significant differences occur at low zenith angles (from 0° to 30°), which is mainly caused by the differences between the upward PCO components. In full dPCCs, severe observational conditions at low elevations are far more visible than those in elevation-only models. Note that low elevation angle signals have more noise, which is caused by crossing through an environment near the surface of Earth. In some horizontal directions and for high zenith angles (from 80° to 90°), the dPCCs obtained by comparison results of calibration one and calibration two exceed 2 mm. For the LEIAR10/NONE antenna, it is accurate in the west and east regions, and for the TRM115000.00/NONE antenna, the most significant differences occur in the north-western and southeastern regions.

Besides the internal repeatability of the experiment itself, external antenna calibration accuracy assessment with other calibration methods is mandatory. For this purpose, available IGS antenna files like igs05.atx, igs08.atx or igs14.atx may be used. These files contain most of the common geodetic absolute antenna datasets calibrated by the other international institutions or research groups. Although the igsxx.atx files contain so called “mean-type” models, they can be used as a good reference for comparison in case of the lack of an individual model. The first approximation of PCV is a mean-type PCC model. If more accurate data are needed for a specific antenna, individual calibration is inevitable [32].

For final external accuracy validation, the final model has been created for both antennas. The final models were created as based on the whole observational window (about five hours), covering three calibration sessions (as a mean result of three individual calibrations). In reference to the calibration values from igs14.atx, assessments similar to Table 2 were performed and are presented in Table 3 (using igs14.atx and Astri/UWM final PCCs models). The assessments in Table 3 demonstrate that the calibrated values for both antennas used in the experiments were estimated correctly and are comparable to IGS mean-type models from the igs14.atx file with an accuracy better than 0.6 mm for the horizontal component and better than 1.5 mm for the vertical component.

**Figure 9:**
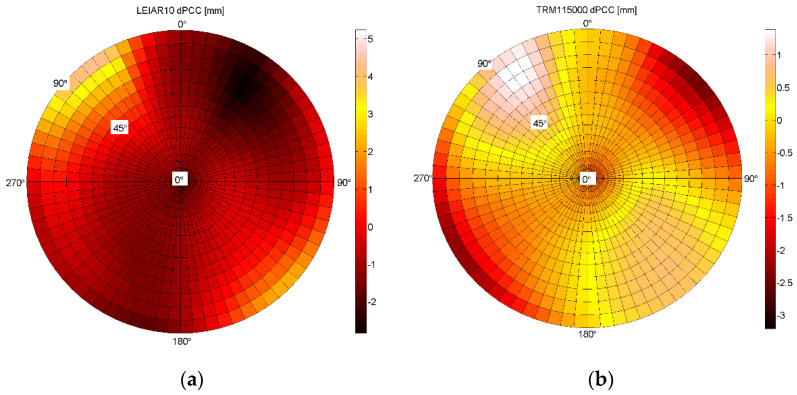
Differences between two independent calibrations of full PCCs for GPS L1 frequency: (**a**) LEIAR10/NONE (left) antenna; (**b**) TRM115000.00/NONE (right) antenna.

Figure 9 presents the differences between IGS mean-type and Astri/UWM final full PCCs models. As previously, comparison was performed using the approach proposed by Schön and Kersten [30].

When analyzing the external accuracy of the achieved results for the full PCC model, it can be seen that the results are slightly worse than in the case of internal accuracy, where the differences exceed 3 mm in some antenna hemisphere regions. This is valid for LEIAR10/NONE, where the differences in northwestern regions at a low elevation exceed 4 mm, and additionally for the TRM115000.00/NONE antenna as well.

As in the case of internal accuracy checking, larger differences (reaching 1.5 mm) occur at low zenith angles (from 0° to 30°), which is mainly caused by the differences in PCO vertical components. Some significant differences of 3–4 mm can be found close to the horizontal signal reception angles (zenith angles from 80° to 90°), possibly caused by the remaining multipath effects and poor antenna receiving performance at low elevations. For the LEIAR10/NONE antenna, such significant differences occur in the northwestern and southeastern hemisphere regions and in the northeastern and southwestern hemisphere regions for the TRM115000.00/NONE antenna.

## 9. Conclusions and Future Work

The paper addresses the initial results of a project aimed at the development and implementation of the field calibration procedure for a multi-frequency and multi-system GNSS. Based on the “Hannover” concept, an absolute antenna phase center calibration algorithm was investigated and a GNSS receiver antenna calibration procedure has been implemented. Robotic arm rotation and tilting scenarios ensured a homogenous observation distribution with regard to the antenna hemisphere, which is critical for high precision antenna phase center parameter estimation.

The analyses of the internal accuracy show that the dPCCs do not exceed 2 mm in the whole antenna hemisphere. The most significant differences occur at low zenith angles (from 0° to 30°), which is mainly caused by the differences in PCO vertical components. Significant differences can also be observed in some azimuthal directions for high zenith angles from 80° to 90°, where dPCCs exceed 2 mm. The reason for the observed phenomenon could be higher noise which is typical for the low elevation angle signals.

When analyzing the external accuracy of achieved results for the full PCCs model, it can be seen that differences are slightly higher than in the case of internal accuracy and exceed 3 mm in some antenna hemisphere regions. As previously described, some larger differences (reaching 1.5 mm) occur at low zenith angles. Some noticeable differences in the order of 3–4 mm can also be found close to horizontal reception angles. Nevertheless, the achieved results demonstrate consistent performance for a Astri/UWM calibration strategy and calibration procedure; however, some aspects (e.g., low elevation PCCs modeling) need to be further investigated in the future.

## Figures and Tables

**Table 1:** Details of calibration campaigns.

**Calibration Date**	
11 September 2020	23 September 2020
**Hardware Used**	
Robot Mitsubishi RV-3S GPS-Reference 2000 external oscillator, Receivers: 2× Topcon Net-G3A Reference antenna: Topcon GR-G3 Calibrated antenna: LEIAR10/NONE	Robot Mitsubishi RV-3S GPS-Reference 2000 external oscillator, Receivers: 2× Topcon Net-G3A Reference antenna: Topcon GR-G3 Calibrated antenna: TRM115000.00/NONE
**Start/End of Calibration Sessions**	
07:41:15/12:44:05	06:55:05/12:03:40

**Table 2:** PCO test solutions for all sessions and the corresponding standard deviations.

Calibration No	Antenna Type	PCO (mm)			PCO SD (mm)		
		North	East	Up	North	East	Up
1	LEIAR10 NONE	+1.31	−0.52	+86.26	0.19	0.25	0.56
2		+1.12	−0.68	+87.33			
3		+0.85	−0.09	+86.05			
igs14.atx		+1.42	−0.25	+88.04	-	-	-
1	TRM115000.00 NONE	+1.03	+0.27	+64.75	0.26	0.22	0.52
2		+0.75	+0.36	+64.05			
3		+0.38	+0.77	+63.49			
igs14.atx		+0.68	−0.12	+65.19	-	-	-

**Table 3:** PCO solutions accuracy compared to IGS mean-type (final PCCs model).

Antenna Type	Igs14.atx MPC (mm)			Astri/UWM MPC (mm)			MPC Differences (mm)		
	N	E	U	N	E	U	ΔN	ΔE	ΔU
LEIAR10 NONE	+1.42	−0.25	+88.04	+1.09	−0.43	+86.55	0.33	0.18	1.49
TRM115000.00 NONE	+0.68	−0.12	+65.19	+0.72	+0.47	+64.10	0.04	0.59	1.09

## Data Availability

Raw data were generated at UWM. Derived data supporting the findings of this study are available from the corresponding author KD on request.
